# Establishment of Heat‐Damaged Model for Hair

**DOI:** 10.1111/jocd.70360

**Published:** 2025-07-30

**Authors:** Yusha Zi, Jianwei Liu, Shuhong Fang, Meng Li, Qing Huang, Xianwu Peng

**Affiliations:** ^1^ Amway (Shanghai) Innovation and Science Co., Ltd. Shanghai China; ^2^ Hangzhou C&K Testing Technic Co., Ltd. Hangzhou China; ^3^ China Pharmaceutical University Nanjing China; ^4^ Amway (China) Co., Ltd. Guangzhou China

**Keywords:** area‐scanning, cuticle morphology, heat‐damaged model, in vitro hair tresses, longitudinal section micrographs, tryptophan content, water distribution

## Abstract

**Background:**

After washing hair, prolonged and frequent use of hair dryers to dry hair can cause heat damage to hair fibers. Currently, various hair care products designed to repair heat damage are available in the market.

**Objective:**

To establish a model of heat‐damaged hair for evaluating the effectiveness of hair care products that claim to repair such damage.

**Methods:**

A blast drying oven was used in this study to expose in vitro hair tresses to 24 h of drying at 60°C, simulating the effects of daily hair drying with a hair dryer for 1 month. The effectiveness of the heat‐damaged model for hair was assessed through microscopic observation and physicochemical testing. Statistical analysis was conducted to compare the cuticle morphology, surface contact angle, water content, mechanical properties, released amount of protein, and tryptophan content of virgin hair, hair dried with a hair dryer over an extended period, and hair from the heat‐damaged model.

**Results:**

Compared to virgin hair, hair dried daily with a hair dryer for the equivalent of 1 month exhibits lifted cuticles. The surface contact angle, water content, mechanical properties, and tryptophan content are significantly reduced, while the released amount of protein is significantly increased. In the heat‐damaged hair model, these indicators are either further worsened or show no significant difference compared to hair dried with a hair dryer for 1 month.

**Conclusion:**

The heat‐damaged model for hair is capable of simulating the damage inflicted on hair by daily use of a hair dryer for a period of 1 month, leading to even more severe damage effects.

## 1 Introduction

The structure of hair fibers, from the center to the periphery, consists of the medulla, cortex, and cuticle, respectively. The cuticle is formed by overlapping layers of cuticle scales [1], which serve multiple functions: They prevent the loss of proteins and moisture from within the hair, maintaining its health and strength; and they reduce friction between the hair and the external environment, minimizing damage, breakage, and splitting. Furthermore, the overlay of cuticle scales prevents external dust and chemicals from penetrating into the hair, effectively shielding the hair from contamination‐induced damage. When the cuticle scales are intact and tightly closed, the hair appears healthy and lustrous; conversely, damaged or incompletely closed cuticle scales result in hair that looks dry and frizzy.

The cuticle scales possess another characteristic: They remain closed and tightly interconnected under dry conditions, but open up when exposed to water [2, 3]. When blow‐drying hair while it is still wet, the hot air passing over the surface of the cuticle scales can cause them to open wider, allowing the moisture inside the hair to be removed more rapidly by the hot air, resulting in hair dehydration and, over time, dryness [4]. The high temperatures and strong winds generated by blow‐drying can also damage the structure of the cuticle scales themselves. Once damaged, the cuticle scales are no longer effective in protecting the hair, and an accumulation of damaged scales can lead to a rough hair surface, increasing friction between hairs and further damaging the hair. Additionally, high temperatures can disrupt the disulfide bonds in hair keratin, causing keratin denaturation. This is the primary reason why frequent use of a blow‐dryer can make hair fragile and prone to breakage. Concurrently, the pigments in hair can also be degraded by high temperatures. When the melanin in hair is damaged, the hair tends to turn yellow.

Common methods for inducing heat damage involve using curling/straightening irons or hair dryers for heat treatment. Echhida et al. [5] heated hair with a curling iron to 180°C for 15 s, followed by a 15‐s cooling period, and repeated this cycle 20, 40, 60, or 80 times to observe changes in the tensile properties of hair fibers. Zhou et al. [6], on the other hand, established a high‐temperature damage model of hair by heating it with a straightening iron for 12 s, followed by a 4‐min cooling period, and cyclically applying this heat–cool interval. They investigated changes in the keratin structure, water content, and cuticle morphology of hair when exposed to temperatures exceeding 200°C. Dussaud et al. [7] even designed an automated flat iron to simulate the perming process. Lee et al. [8] compared changes in the cuticle morphology, water content, and color of hair after natural drying at room temperature and after blow‐drying 30 times at temperatures of 47°C, 61°C, or 95°C. All the aforementioned methods suffer from inaccuracies in temperature control and uneven heat distribution on the hair, which affect the stability and reproducibility of test results. In this study, a heat‐damaged hair model established using a blast drying oven ensured stable temperature and uniform heat distribution on the hair, providing a scientific basis for evaluating the effectiveness of hair care products designed to repair heat damage.

## 2 Materials and Methods

### 2.1 Hair Samples

Virgin black Asian hair (15 cm in length, 4 cm in width, and the weight is 5 g) was provided by Shanghai Jiaze Trading Co. Ltd. (Shanghai, China). Before using, the hair tresses were washed using a 10% (w/w) sodium lauryl sulfonate (SLS; Macklin, Shanghai, China) solution, with subsequent drying under ambient conditions (temperature of 20°C–22°C, relative humidity of 40%–60%) for 24 h.

The dried virgin hair tresses were wetted with water and then dried using an EH‐WNE6A hair dryer (Panasonic Wanbao, Guangdong, China). The hair tresses were suspended 10 cm away from the hair dryer nozzle and blown on the highest setting for 3 min on each side. The dried hair tresses were subsequently rewetted and dried again following the aforementioned procedure. This wet‐and‐dry cycle was repeated 30 times, equivalent to daily use of the hair dryer for drying hair over the course of 1 month. (The term “treated hair” is hereinafter used for hair tresses that have been repeatedly wetted and then blow‐dried 30 times as per the above description).

### 2.2 Establishment of Heat‐Damaged Model for Hair

To establish a heat‐damaged model, the DGG‐9123A electrically heated constant temperature blast drying oven (Senxin, Shanghai, China) was utilized. Based on the temperatures at the nozzle of a hair dryer set to its three different settings, the oven temperatures were respectively set to 40°C, 60°C, and 90°C. Virgin hair tresses were exposed to these conditions for 6, 12, and 24 h to screen and establish the optimal conditions for the heat‐damaged model for hair.

### 2.3 Microscopic Observation of the Cuticle Morphology

The cuticle morphology was investigated by a S‐3400 N scanning electron microscope (SEM, Hitachi, Tokyo, Japan). A 1‐cm length of hair fiber was swiftly sectioned off 5 cm from the hair tip using a blade, then fixed onto the stage with double‐sided adhesive tape, coated with conductive paste, and sputter‐coated with a layer of Au.

The longitudinal sections of hair fibers were investigated by an AE31 EF‐INV inverted fluorescence microscope (Motic, Fujian, China). Hair fibers were embedded in paraffin wax (Ctiotest, Jiangsu, China) and sectioned using a HistoCore MULTICUT rotary cutting machine (Leica Microsystems, Shanghai, China). The hair fiber sections were then transferred onto glass slides, dried, and stained with hematoxylin–eosin for 5 min. Excess stain was rinsed off with distilled water. The paraffin was dissolved using xylene, and after allowing the preparations to stand for 12 h, observations were conducted.

### 2.4 Surface Contact Angle Test

The surface contact angle was measured using a DSA100 contact angle tester (A. Krüss Optronic, Hamburg, Germany), with deionized water as the contact liquid. The hair fibers were secured above the sample stage, and the volume of each droplet dispensed was set to 2 μL. Deionized water droplets were then placed onto the hair fibers, and the surface contact angle test results were recorded.

### 2.5 Water Content Test

The water content of hair fibers was analyzed using a TGA 4000 thermal gravimetric analyzer (PerkinElmer, Massachusetts, USA), under a nitrogen atmosphere. A 20 mg sample of hair fibers was placed into a crucible. The initial temperature was set at 25°C, and then, it was increased to 65°C at a rate of 20°C/min, held for 8 min, and subsequently increased to 180°C at a rate of 20°C/min, held for 20 min.

In order to investigate the distribution of water within the cross section of hair, hair fibers were embedded in paraffin wax. Subsequently, sectioning was employed, and the sections were analyzed by the Spotlight 400 Fourier transform infrared spectrometer (FTIR) imaging system (PerkinElmer). The FTIR images were recorded with a spatial resolution of 1.5 μm and a spectral resolution of 4 cm^−1^. The spectra were recorded in the 1450 cm^−1^.

### 2.6 Mechanical Properties Test

The single‐fiber mechanical properties of the hair tresses were measured by a Fibra.one multifunctional hair tress testing instrument (Dia‐Stron, London, UK) with the stress accessory. For each group of the treated hair strands, 30 single hair fibers with diameters of 90–110 μm were selected from the strands under the microscope [9], characterizing the mechanical properties of hair fibers using break total work done.

### 2.7 The Released Amount of Protein Test

To quantify the released amount of protein in human hair, the bicinchoninic acid (BCA) assay was applied, using 0.200 g of hair samples cut into snippets, copper‐BCA reagent (Weiyel, Beijing, China), and distilled water. Also, to calibrate the quantification curve, bovine's albumin (Cato, Guangdong, China) was used as the standard reference. The chopped hair and 5 mL of distilled water were placed inside a reaction tube, subjected to shaking for 4 h, followed by extraction at 48°C–52°C for 18 h, with this process being repeated three times. Subsequently, each sample solution was removed and centrifuged for 10 min. A 1 mL aliquot of the upper turbid suspension was transferred to a colorimetric tube, and 5 mL of copper‐BCA reagent was added immediately, followed by thorough mixing. After 30 min, the absorbance values of each sample solution were measured at 562 nm in the UV‐1600PC Spectrophotometer (Mapada, Shanghai, China). The released amount of protein in the hair samples was calculated according to Equation (1).

(1) $$\omega_{1}=\frac{\rho \cdot V}{m}$$

where *ω*_1_ is the released amount of protein; *m* is the mass of the hair sample; *ρ* is the mass concentration of protein in the sample solution, calculated by substituting into the quantification curve; and *V* is the constant volume.

### 2.8 Tryptophan Content Test

To quantify the tryptophan content in human hair, the LC‐20 ad high‐performance liquid chromatography (HPLC; Shimadzu, Kyoto, Japan) equipped with a fluorescence detector (FLD) was utilized. The hair tresses were degreased using petroleum ether through ultrasonic treatment for 30 min. The chopped hair (0.0150 g of hair samples, cut into snippets) was placed into a hydrolysis tube. Three milliliter of freshly prepared hydrolysis solution (5.5 mol/L sodium hydroxide solution containing 0.5% mass fraction of soluble starch) was added. The mixture was then subjected to vacuum hydrolysis at 110°C for 20 h. The hydrolysate was transferred to a 25‐mL volumetric flask containing hydrochloric acid and rinsed with distilled water 5–6 times. The pH of the solution was adjusted to 7–8 using sodium hydroxide solution, and the volume was made up to the mark with distilled water and prepared for analysis. Also, to calibrate the quantification curve, L‐tryptophan (Changqing, Zhejiang, China) was used as a standard reference. Liquid chromatography conditions: An ODS‐3 C_18_ column (4.6 mm × 250 mm, 5 μm) was used; the mobile phase was a mixture of sodium acetate (0.0085 mol/L, pH = 4) and methanol in a ratio of 90:10; the flow rate was set at 1.0 mL/min; the column temperature was maintained at 30°C; the injection volume was 10 μL; and the excitation wavelength was set at 283 nm with an emission wavelength of 343 nm. L‐tryptophan standard solutions were injected separately to construct a standard curve. The solution to be tested was then injected, and the mass concentration of tryptophan in the solution was determined based on the standard curve. The tryptophan content in the hair samples was calculated according to Equation (2).

(2) $$\omega_{2}=\frac{\rho \cdot V}{m}$$

where *ω*_2_ is the tryptophan content; *m* is the mass of the hair sample; *ρ* is the mass concentration of tryptophan in the sample solution, calculated by substituting into the quantification curve; and *V* is the constant volume.

### 2.9 Statistical Data Analysis

The results of the calculations are expressed as mean ± SD. Data statistical analysis was conducted using SPSS Statistics 25.0. Comparisons between groups were performed using a two‐independent‐sample test with a two‐tailed test and a significance level of *α* = 0.05.

Significance notations: “ns” indicates *p* ≥ 0.05, indicating no significant difference; “*” indicates 0.01 ≤ *p* < 0.05, indicating a significant difference; and “**” indicates *p* < 0.01, indicating a highly significant difference.

## 3 Results

### 3.1 Heat‐Damaged Model for Hair

**FIGURE 1:**
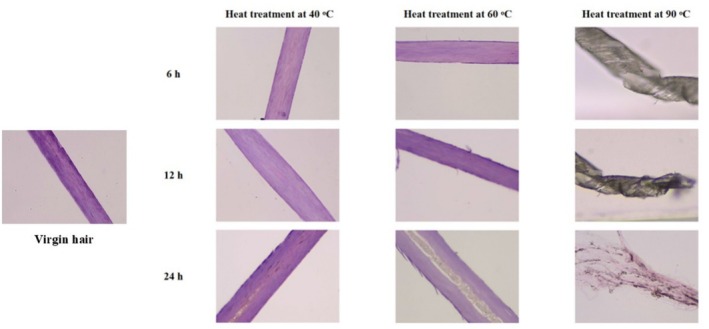
Micrographs of hair fiber status.

As shown in Figure 1, after heat treatment at 40°C, the cuticle morphology showed almost no difference compared to the untreated virgin hair, with only mild lifting observed in the cuticles of hair that was treated for 24 h. After heat treatment at 60°C for 6 h, individual hair cuticles were observed to lift up. As the duration of heat treatment increased at this temperature, the number of lifted hair cuticles also increased. After heat treatment at 90°C, followed by embedding and sectioning, microscopic observations revealed that the hair fibers were completely fragmented.

As shown in Table 1, compared to treated hair, hair that has undergone heat treatment at 40°C exhibits a highly and significantly greater break total work done (16.82 ± 3.34 J, 15.93 ± 5.30 J, and 15.89 ± 4.77 J, respectively), with a highly significant difference (*p* < 0.01). The tensile break work of hair subjected to heat treatment at 60°C for 6 h is 15.11 ± 3.43 J, which exhibits a highly significant difference compared to treated hair (*p* < 0.01). At this temperature, as the treatment duration increases, the fracture work decreases to 12.20 ± 2.38 J at 24 h, showing no significant difference compared to treated hair (*p* ≥ 0.05). The break total work done of hair fibers to heat treatment at 90°C is lower than that of treated hair (12.01 ± 2.97 J, 11.67 ± 2.17 J, and 11.20 ± 2.16 J, respectively); there is no significant difference compared to treated hair (*p* ≥ 0.05).

**TABLE 1:** Break total work done test results after heat treatment.

	Virgin hair	Treated hair	Heat treatment at 40°C			Heat treatment at 60°C			Heat treatment at 90°C		
			6 h	12 h	24 h	6 h	12 h	24 h	6 h	12 h	24 h
Break total work done (J)	17.15 ± 2.66	12.42 ± 2.13	16.82 ± 3.34	15.93 ± 5.30	15.89 ± 4.77	15.11 ± 3.43	14.13 ± 3.07	12.20 ± 2.38	12.01 ± 2.97	11.67 ± 2.17	11.20 ± 2.16
Significance	**		**	**	**	**	*	ns	ns	ns	ns

*Note:* Conduct significance analysis with the treated hair.

Significance: ns *p* ≥ 0.05; * 0.01 ≤ *p* < 0.05; ** *p* < 0.01.

As presented in Table 2, the released amount of protein of hair subjected to heat treatment at 40°C is significantly lower than that of treated hair (1.31 ± 0.01, 1.32 ± 0.01, and 1.32 ± 0.00 mg/g, respectively, *p* < 0.01). The released amount of protein of hair subjected to heat treatment at 60°C for 6 h was 1.37 ± 0.02 mg/g, which was significantly lower than that of the treated hair (*p* < 0.01); when the heat treatment duration was extended to 24 h, the released amount of protein increased highly and significantly to 1.68 ± 0.03 mg/g compared to the treated hair (*p* < 0.01). The released amount of protein of hair subjected to heat treatment at 90°C is significantly greater than that of the treated hair (2.09 ± 0.02, 2.11 ± 0.03, and 2.08 ± 0.04 mg/g, respectively, *p* < 0.01). (The term “damaged hair” is hereinafter used for hair tresses that have been placed in the blast drying oven at 60°C for 24 h as per the above description).

**TABLE 2:** The released amount of protein test results after heat treatment.

	Virgin hair	Treated hair	Heat treatment at 40°C			Heat treatment at 60°C			Heat treatment at 90°C		
			6 h	12 h	24 h	6 h	12 h	24 h	6 h	12 h	24 h
The released amount of protein (mg/g)	1.27 ± 0.03	1.53 ± 0.03	1.31 ± 0.01	1.32 ± 0.01	1.32 ± 0.00	1.37 ± 0.02	1.54 ± 0.04	1.68 ± 0.03	2.09 ± 0.02	2.11 ± 0.03	2.08 ± 0.04
Significance	**		**	**	**	**	ns	**	**	**	**

*Note:* Conduct significance analysis with the treated hair.

Significance: ns *p* ≥ 0.05; * 0.01 ≤ *p* < 0.05; ** *p* < 0.01.

### 3.2 The Cuticle Morphology

**FIGURE 2:**
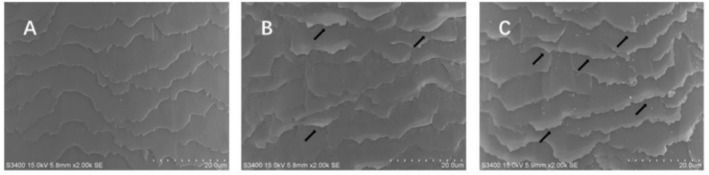
SEM micrographs of cuticle morphology of hair fiber surface. (A) Virgin hair. (B) Treated hair. (C) Damaged hair.

The SEM was employed to assess the cuticle morphology of hair fibers, with the results shown in Figure 2. Figure 2A shows the cuticles of virgin hair exhibit an overlapping arrangement, with intact structures and smooth edges. Figure 2B,C indicates where severe damage occurred in the cuticles of hair fibers, caused by the heat treatment, as the arrows highlight. Lifted or even detached cuticles of treated and damaged hair can be observed, with some cuticles fragmented and edges roughened.

**FIGURE 3:**
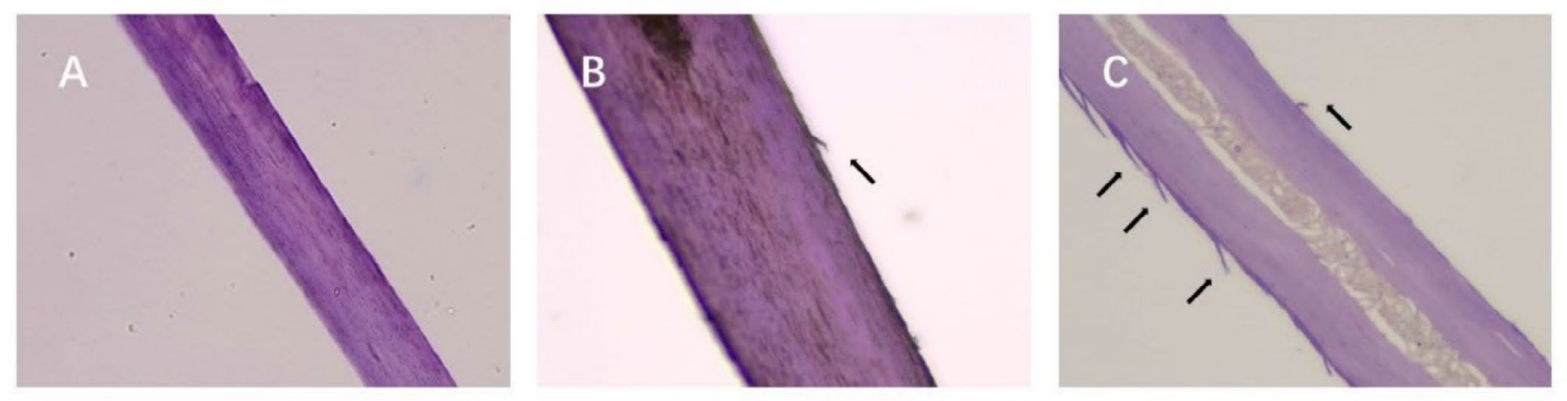
Longitudinal section micrographs of cuticle morphology of hair fiber surface. (A) Virgin hair. (B) Treated hair. (C) Damaged hair.

Figure 3 presents the microscopical examination of the longitudinal section of the hair, conducted to observe the morphology of the cuticles more clearly. Figure 3A shows no lifting of the cuticles in the virgin hair. As depicted in Figure 3B,C, the arrows indicate where severe damage occurred in the cuticles of hair fibers, caused by the heat treatment. Similarly, the condition observed in damaged hair is more severe than that in treated hair.

### 3.3 Surface Contact Angle

When measuring the wetting effect of a liquid on a solid surface, the surface contact angle needs to be considered. In this study, the surface contact angle refers to the angle between the tangent to the edge where the liquid droplet is in contact with the hair fiber and the plane of the hair fiber when the liquid droplet is placed on the hair fiber. Table 3 presents the contact angle test results for virgin hair, treated hair, and damaged hair. Additionally, visual images have been incorporated into the table to clearly demonstrate their hydrophilic and hydrophobic properties through observation.

**TABLE 3:** Test result of surface contact angle.

	Virgin hair	Treated hair	Damaged hair
Surface contact angle (°)	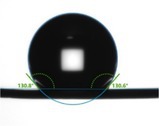 129.64 ± 5.83	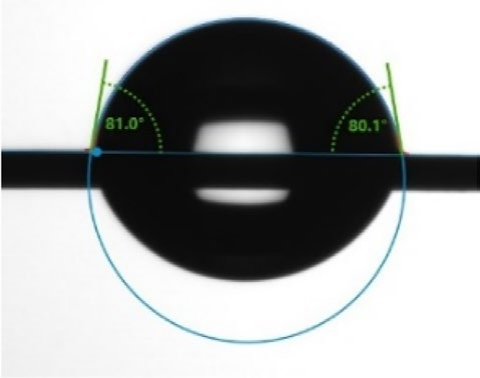 92.39 ± 9.89	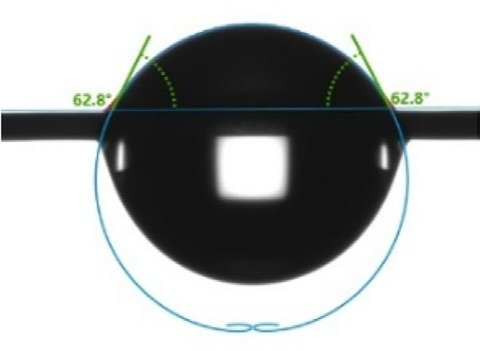 63.76 ± 1.31

Specifically, if the surface contact angle is < 90°, the hair fiber surface exhibits hydrophilic behavior; if the surface contact angle exceeds 90°, it displays hydrophobic behavior; and when the surface contact angle surpasses 150°, the hair fiber demonstrates superhydrophobic properties [10]. The surface contact angle of the virgin hair was 129.64° ± 5.83°, exhibiting hydrophobicity. The surface contact angle of the treated hair decreased highly significantly to 92.39° ± 9.89° (*p* < 0.01), remaining hydrophobic. Notably, the surface contact angle of the damaged hair decreased highly significantly compared to the treated hair (*p* < 0.01), reaching 63.76° ± 1.31°.

### 3.4 Water Content

**FIGURE 4:**
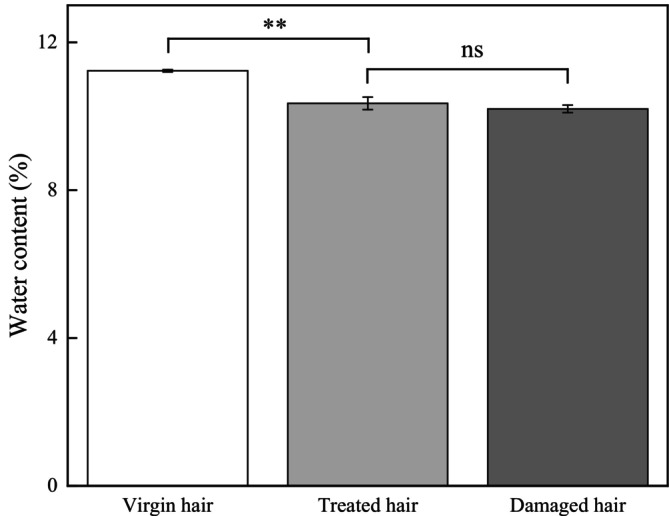
Test result of water content.

Figure 4 shows that virgin hair exhibited a water content of 11.23% ± 0.03%, which is consistent with previous literature reports [11], whereas treated hair demonstrated a highly significant decrease to 10.35% ± 0.17% (*p* < 0.01). Notably, the water content of damaged hair, at 10.20% ± 0.10%, did not significantly diverge from that of the treated hair (*p* ≥ 0.05).

**FIGURE 5:**
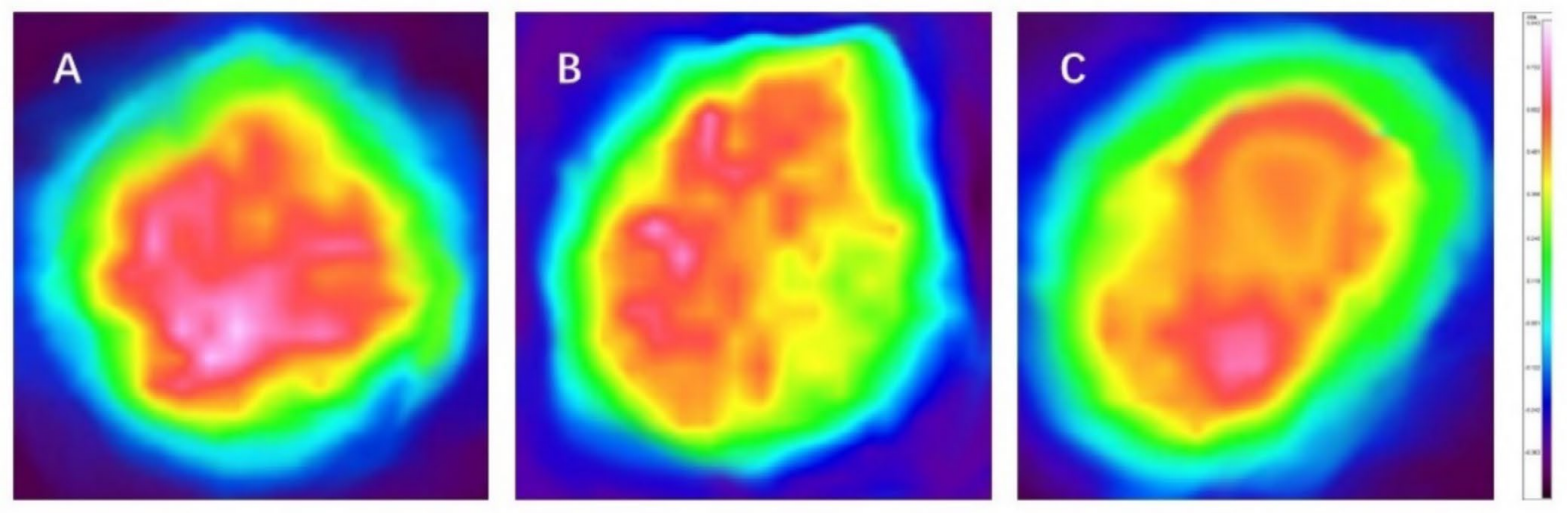
Area‐scanning regions located on the cross section of hair fibers. (A) Virgin hair. (B) Treated hair. (C) Damaged hair.

Figure 5 shows that an area‐scanning process was performed on regions of the cross sections of hair fibers. The O–H bond stretching vibration of water molecules occurs at 1450 cm^−1^. A redder color indicates a higher water content, while a blue color signifies a lower water content. As shown in Figure 5A, the cortex of virgin hair has a high and relatively uniform water content. As shown in Figure 5B,C, the reduced red areas in treated and damaged hair indicate lower water content, respectively, in agreement with the thermogravimetric analysis results. Additionally, Figure 5C demonstrates a reduced red area in damaged hair relative to treated hair, implying a further decrease in water content.

### 3.5 Mechanical Properties

**FIGURE 6:**
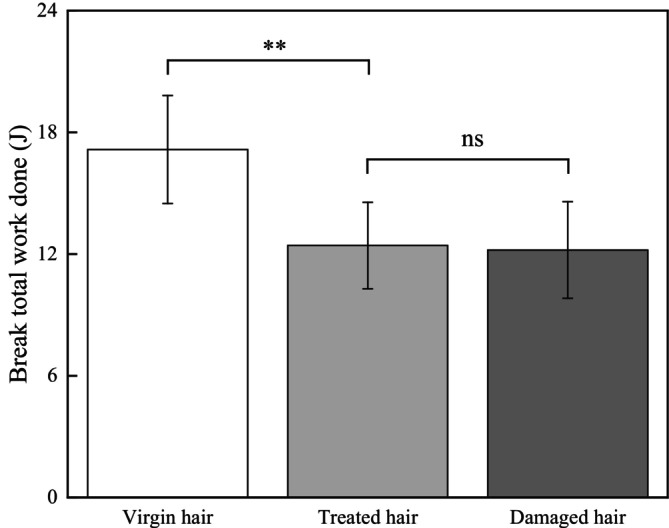
Test result of the break total work done.

As shown in Figure 6, the break total work done of virgin hair was 17.15 ± 2.66 J, which highly significantly decreased to 12.42 ± 2.13 J (*p* < 0.01) for treated hair. While the break total work done of damaged hair (12.20 ± 2.38 J, *p* ≥ 0.05) showed no significant difference compared to that of treated hair.

### 3.6 The Released Amount of Protein

**FIGURE 7:**
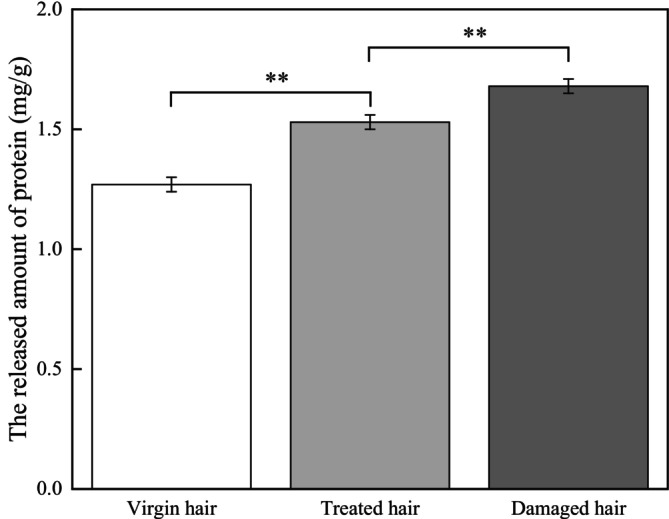
Test result of the released amount of protein.

As shown in Figure 7, damaged hair becomes more fragile, and this characteristic can be utilized to quantitatively assess the degree of damage during heat damage processes based on the amount of protein released under mechanical oscillation. The released amount of protein for virgin hair was 1.27 ± 0.03 mg/g. For treated hair, the released amount of protein significantly increased to 1.53 ± 0.03 mg/g (*p* < 0.01). Compared to treated hair, the released amount of protein for damaged hair showed a highly significant increase to 1.68 ± 0.03 mg/g (*p* < 0.01).

### 3.7 Tryptophan Content

**FIGURE 8:**
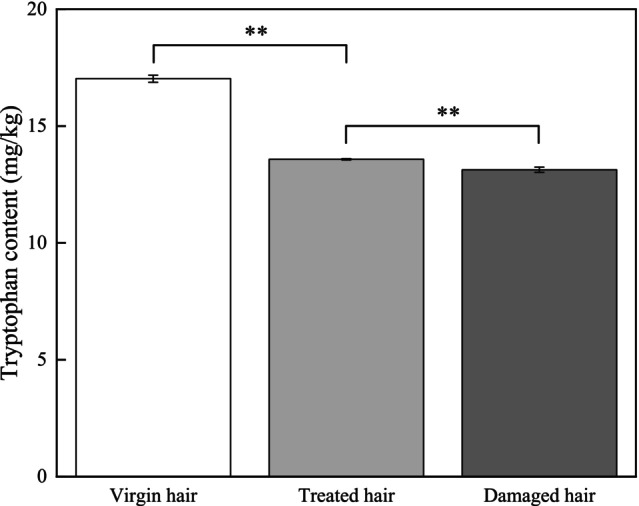
Test result of tryptophan content.

Data in Figure 8 show that the tryptophan content in virgin hair was 17.03 ± 0.15 mg/kg, while the tryptophan content in treated hair highly significantly decreased to 13.58 ± 0.03 mg/kg (*p* < 0.01), and the tryptophan content in damaged hair was 13.13 ± 0.11 mg/kg, showing a highly significant difference compared to the treated hair (*p* < 0.01; Figure 8).

## 4 Discussion

This study developed and validated a method for establishing a hair heat‐damaged model using in vitro hair tresses to assist in assessing the efficacy of hair care products designed to repair heat damage. To this end, a blast drying oven was utilized to simulate the prolonged and frequent use of hair dryers for drying hair. The effectiveness of the model was demonstrated through statistical evaluation of the microstructure and physicochemical properties of virgin hair, treated hair, and damaged hair using in vitro hair tresses. It has been proven that the heat‐damaged model can simulate the daily use of hair dryers for drying hair over a month, resulting in more severe damage effects.

The hair tresses were placed in the 90°C blast‐drying oven for 6 h. Upon removal, the hair fibers emitted a burnt odor, and after embedding and sectioning, they were completely fragmented (Figure 1), rendering them unusable for subsequent research. After undergoing heat treatment at 40°C, the break total work done of the hair fibers was greater than that of the treated hair, with a highly significant difference observed (*p* < 0.01; Table 1). The mechanical properties of the hair fibers under these conditions were superior to those of the treated hair, yet this scenario failed to simulate the effects of continuous hair dryer use for 1 month. After heat treatment at 60°C for 6 h, the released amount of protein of hair fibers was lower than that of the treated hair, suggesting that these conditions could not simulate the effects of continuous hair dryer use for 1 month. When the heat treatment was extended to 12 h at 60°C, there were no significant differences in the released amount of protein compared to the treated hair (*p* ≥ 0.05). However, upon heat treatment at 60°C for 24 h, the released amount of protein increased highly significantly (*p* < 0.01; Table 2), indicating a greater degree of damage to the hair fibers compared to the treated hair.

To sum up, the blast drying oven temperature was set to 60°C, and the virgin hair tresses were placed inside for 24 h, simulating the effect of daily hair drying with an electric blow‐dryer over the course of 1 month.

The cuticles of virgin hair exhibit no lifting (Figures 2A and 3A). Compared to the treated hair, the lifting of cuticle scales is more severe in damaged hair (Figures 2B,C and 3B,C). Once the cuticles were damaged, heat can reach the cortex, and the hair may break easily (Figure 6) since there was no protection for the cortex. The results of cuticle morphology indicate that the heat‐damaged model is capable of simulating the continuous use of a hair dryer for a period of 1 month, resulting in more severe damage effects. The virgin hair exhibits hydrophobicity, which is consistent with previous literature reports [12]. The contact angle of damaged hair decreased significantly (*p* < 0.01), exhibiting hydrophilicity (Table 3). This reduction can be attributed to the decomposition of the hydrophobic protective layer on the outermost surface of the hair fibers due to heat treatment, resulting in alterations to the hair's hydrophobicity. The surface contact angle results suggest that the heat‐damaged model is capable of simulating the continuous use of a hair dryer for a period of 1 month, leading to more severe damage effects. Heat treatment alters the structure of hair fibers, resulting in damage that adversely affects the hair's water absorption capacity under ambient humidity conditions, thereby diminishing its moisture‐retention and water‐uptake properties [13]. In comparison with virgin hair, damaged hair exhibits a substantial and highly significant reduction in water content (*p* < 0.01; Figure 4), along with a decreased water distribution in the cortex (Figure 5). The water content results demonstrate that the thermal damage model can simulate the effects of daily blow‐dryer use for a month, leading to more severe damage. Various types of damage all contribute to a reduction in the mechanical properties of hair [14, 15]. After damage to the cortex of hair (Figures 2 and 3), its mechanical strength decreases (Figure 6), making it prone to breakage [16]. The results of mechanical properties indicate that the heat‐damaged model is capable of simulating the daily use of a hair dryer for drying hair over a 1‐month period. Hair fibers are primarily composed of keratin. When hair undergoes damage, the outermost barrier is lost first, followed by gradual damage to the various tissue structures within the cortical layer. A higher released protein amount indicates more severe hair damage (Figure 7). In their study on oxidative damage to hair, Barba et al. [17] found that hair treated with antioxidants exhibited less released protein amount after oxidative damage compared to untreated hair. The results of the released protein amount indicate that the heat‐damaged model can simulate the daily use of a hair dryer for drying hair over a 1‐month period, resulting in more severe damage effects. Furthermore, the BCA assay method exhibits high sensitivity, simplicity in operation, and minimal interference from extraneous substances. Additionally, the reagents and the color complexes they form demonstrate excellent stability, making it a superior choice compared to the Bradford method and Lowry method [18, 19]. The tryptophan content in hair can serve as a sensitive marker for photodamage to hair [20]. In this study, the tryptophan content in damaged hair is significantly lower than that in virgin hair; this suggests that the more severe the damage to the hair fibers, the lower the tryptophan content. Jachowicz et al. [21] conducted a study using fluorescence spectroscopy and found that the more severe the hair damage, the lower the tryptophan content. The results of tryptophan content indicate that the thermal damage model can simulate the daily use of a hair dryer for drying hair over a period of 1 month, leading to more pronounced damage effects.

Compared to virgin hair, both treated hair and damaged hair exhibited lifted cuticle morphology, decreased surface contact angles, reduced water content, degraded mechanical properties, increased released amount of protein, and decreased tryptophan content. Notably, these indicators were worse or showed no significant difference in damaged hair compared to treated hair. The effectiveness of the heat‐damaged model for hair has been demonstrated.

## Author Contributions

Yusha Zi: responsible for the development of the research proposal. Jianwei Liu: responsible for the development and consultation of in vitro tests; contributed to paper editing. Shuhong Fang: contributed to cosmetic efficacy testing and paper editing; Meng Li: contributed to paper editing; established a model of heat‐damaged hair. Qing Huang: responsible for the development and consultation of in vitro tests; evaluated the model of heat‐damaged hair. Xianwu Peng: the initiator and sponsor of the research.

## Conflicts of Interest

The authors declare no conflicts of interest.

## Data Availability

The authors have nothing to report.
